# Alzheimer’s Disease Pathogenic Mechanisms: Linking Redox Homeostasis and Mitochondria-Associated Metabolic Pathways Through Nuclear Factor Erythroid 2-Related Factor 2 (Nrf2)

**DOI:** 10.3390/antiox14070812

**Published:** 2025-07-01

**Authors:** Agueda Rostagno, Jorge Ghiso

**Affiliations:** Department of Pathology, New York University Grossman School of Medicine, 550 First Avenue, New York, NY 10016, USA

**Keywords:** Alzheimer’s disease, oxidative stress, mitochondria, Nrf2, antioxidant therapies

## Abstract

Alzheimer’s disease (AD) is the leading cause of dementia, with a prevalence expected to escalate with the aging of the world population as life expectancy increases. In spite of significant progress made in the investigation of the etiology and pathogenesis of the disease, many mechanistic aspects that could support the implementation of novel therapeutic avenues remain unresolved. Research during the last decade has revealed a crucial role for mitochondria-mediated pathways dysregulation as significant contributors to the disease, highlighting the relevance of changes in brain metabolism and bioenergetics as well as the induction of oxidative stress conditions for neurodegeneration. This review summarizes mitochondrial functional changes associated with AD with emphasis in the dysregulation of redox homeostasis and the role of nuclear factor erythroid 2-related factor 2 (Nrf2), not only as a central regulator of the antioxidant response but also as a more recently described modulator of cellular metabolic pathways. Potential therapeutic strategies targeting oxidative stress and mitochondrial dysfunction are also discussed, with particular emphasis on the use of small molecules Nrf2 activators. Exploiting the multifactorial properties of the transcription factor in either novel or combination-based pharmacological approaches targeting multiple genes and pathways may contribute to providing more definitive and precise therapeutic perspectives.

## 1. Introduction

Alzheimer’s disease (AD) is the most common type of dementia, accounting for 60% to 80% of all cases. Currently, it is estimated that the disease affects about 50 million patients worldwide, with 10 million new cases reported annually [1]. Only a small percentage of AD cases are associated with dominant genetic mutations in the amyloid precursor protein (APP) and the presenilin (PSEN) genes, while the vast majority of the cases are sporadic in nature and have no single genetic cause [2,3]. In spite of the absence of genetic linkages for most cases, aging and the presence of the Ꜫ4 allele of the apolipoprotein E gene are considered significant risk factors for the disease [3]. In addition, environmental and metabolic factors such as diabetes, cerebrovascular disease, poor diet, head injury, and stress are typically associated with an increased risk of the disease [4]. The deposition of amyloid-β (Aβ) in brain parenchyma and cerebral vasculature, together with the presence of intraneuronal neurofibrillary tangles and the gradual loss of synapsis, are central neuropathological hallmarks of AD [2]. The transition of Aβ from a soluble monomeric proteoform normally present in body fluids to oligomeric, protofibrillar, and endpoint fibrillar structures is currently considered a significant contributor to the disease pathogenesis. These transitions involve the formation of multiple heterogeneous structures with dissimilar solubility and molecular mass that may exert different impacts on disease pathogenesis. Over the years, different species—among them Aβ-derived diffusible ligands and protofibrils, as well as small and large oligomers—have demonstrated detrimental effects in vivo and in vitro, ranging from neuronal toxicity, neuroinflammation, and tau hyperphosphorylation to long-term potentiation impairment and memory deficits [5]. Indeed, multiple lines of evidence currently support a role for oligomeric forms of Aβ in AD pathophysiology [6,7], exerting potent toxic effects on neurons that result in synaptic alterations and cell death [7,8,9,10]. Further support for prefibrillar structures as key elements in the molecular pathogenesis of AD stems from immunization data in animal models—in which specific anti-Aβ antibodies targeting these intermediate species restore memory function [11,12]—and in humans, where passive immunization with various monoclonal antibodies in clinical trials either resulted in the first FDA approved therapy for AD or are currently being evaluated [9,13,14].

Despite the more than 100 years that has passed since the discovery of the disease, the complex molecular mechanisms leading to AD pathophysiology are still not fully elucidated. Among the different pathways affected by the disease, a central role for mitochondrial detrimental changes has been described at early stages of the disease [15,16,17,18,19,20,21,22]. Indeed, subjects with mild cognitive impairment, who do not yet exhibit amyloid deposition or the presence of neurofibrillary tangles show signs of oxidative stress, as evidenced by enhanced protein oxidative modifications [23,24] and increased lipid peroxidation [22]. These changes coexist with significantly decreased levels of the endogenous antioxidant systems superoxide dismutase (SOD) and catalase, as well as reduced expression of the glutathione reductase/glutathione peroxidase ratio [25,26,27]. The mitochondrial change translates into changes in the organelle function and bioenergetics, not only in neurons but also in astrocytes and in cells of the cerebral microvasculature [28,29,30,31,32,33,34,35,36,37,38,39,40,41,42,43,44], overall supporting a causative role for these abnormalities in AD [18,45]. Mitochondria not only exert central control of cell bioenergetics, but they are also the most important generators of oxygen and nitrogen derived free radicals, collectively termed reactive oxygen and nitrogen species (ROS and RNS) [46]. Reduced ATP production, oxidative stress, impairment in cerebral glucose metabolism, downregulation of central elements for oxidative phosphorylation (OxPhos), changes in anaplerotic enzymes, and synaptic alterations have been reported in vivo in AD brains [47,48,49] and several of these features have been recapitulated in vitro with oligomeric Aβ assemblies [29,30,31,32,50,51,52] and in transgenic models of Aβ deposition [53,54,55,56,57,58,59,60,61,62,63,64,65,66,67,68,69,70]. Whether these events result from the disruption of a particular molecular mechanism or are the consequence of multiple affected pathways acting independently or synergistically is not entirely clear. This review focuses on the disturbance of redox homeostasis and its contribution to AD pathogenesis, highlighting the role of the transcription factor nuclear erythroid 2-related factor 2 (Nrf2) on the antioxidant response element (ARE) pathway, as well as on various metabolic aspects of AD pathobiology.

## 2. Brain Energy Requirements and the Essential Role of Mitochondria

In spite of representing only about 2% of the body weight in humans, the brain is responsible for approximately one quarter of the total oxygen and glucose consumption of the organism [71,72]. This extraordinary energy requirement is crucial to maintain ion gradients across neuronal plasma membranes that are critical for generation of action potentials, and for sustaining transport systems at endothelium barriers [73,74]. About 90% of the energy needs in the brain are generated through glucose oxidation [75,76], a carefully controlled process leading to high yield ATP production [77,78]. Since the brain stores little energy, mitochondria are crucial for maintaining the elevated energy requirements necessary to avoid cellular dysfunction and death [77,78]. These highly dynamic organelles control cell bioenergetics and ROS homeostasis, performing critical functions for the cell that extend beyond simply participating in ATP synthesis. In addition to energy production, mitochondria play vital roles as critical regulators of neuronal cell death and survival, modulating apoptosis, ferroptosis, and inflammasome activation, with new research supporting an important role in the modulation of blood–brain barrier (BBB) function as well as in the regulation of synaptic integrity and the underlying mechanisms of learning and memory [18,79,80,81,82].

At present, many studies using cell lines and mouse models have provided compelling evidence indicating a significant contribution of mitochondrial dysfunction to the etiology of Aβ-related AD pathology [83,84]. Changes in the number and morphology of mitochondria, alterations in the organelles’ intracellular transport, reduced cytochrome oxidase activity, modifications in the mitochondrial membrane potential, and increased oxidative stress have been reported in the disease [82,84,85,86]. Neurons, in particular, are highly dependent on mitochondria, organelles that accumulate at synapses, helping to power their high metabolic demand and leading to a high level of ROS production at these sites. This generation of reactive molecules, in conjunction with insufficient levels of antioxidants, often leads to oxidative stress [87], as typically observed during aging, which is the primary risk factor for neurodegenerative diseases. Indeed, it is considered today that the chronic accumulation of oxidative damage to lipids, DNA, and proteins by reactive oxygen and nitrogen species are significant contributors to disease pathogenesis. The brain in particular exhibits high susceptibility to ROS formation, as it is rich in polyunsaturated fatty acids—the preferred substrates of these reactive species—while showing a low expression of antioxidant systems [20,88,89,90]. These features strengthen the relevance of oxidative stress mechanisms as contributing factors to the pathogenesis of neurodegenerative diseases [87,88,91,92].

## 3. Maintaining Cell Redox Homeostasis

Redox imbalance between oxidants and antioxidants leads to oxidative stress and is a crucial contributor to many pathological conditions, including cardiovascular diseases and neurodegenerative conditions. Although redox-related disorders may also originate from the production and dysregulation of reactive sulfur species, this review will focus on the mechanisms of production and regulation of reactive oxygen and nitrogen species, most of which are unstable free radicals containing unpaired electrons in the outer shell.

### 3.1. Generation and Regulation of Reactive Oxygen Species

**Figure 1:**
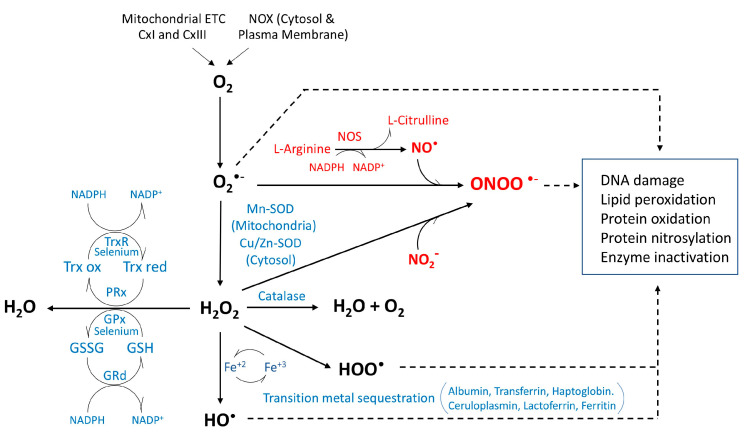
Generation and homeostatic regulation of reactive oxygen and nitrogen Species. The anion superoxide (O_2_^•−^) together with hydrogen peroxide (H_2_O_2_) are the most common reactive oxygen species (ROS). Their generation is endogenously regulated at various levels to restrict their harmful activity. Superoxide dismutases, either mitochondrial (Mn-SOD) or cytosolic (Cu/Zn-SOD), decompose the anion superoxide into the less reactive H_2_O_2_, which in turn can be further transformed in water and molecular oxygen by the action of catalase. Two additional thiol redox mechanisms with unknown functional interaction among themselves are also involved in the degradation of H_2_O_2_: (a) the thioredoxin reductase (TrxR), with the capability to reduce oxidized thioredoxins (Trx ox); and (b) the glutathione reductase (GRd), with the ability to reduce oxidized glutathione (GSSG) and generate reduce glutathione (GSH). Both reductases use NADPH as the source of reducing equivalents while the pertinent reverse reactions, generating the oxidized forms of the thiol switch proteins, are catalyzed by peroxiredoxins (PRx) and glutathione peroxidase (GPx), respectively. H_2_O_2_ in the presence of transition metals (e.g., Fe^2+^) is able to generate the hydroxyl (HO^•^) and the hydroperoxide (HOO^•^) radicals, which can be controlled through transition metal sequestration by several plasma proteins. Reactive nitrogen species (RNS), like peroxynitrite (ONOO^•−^), can be formed by the reaction of anion superoxide with nitric oxide (NO^•^)—generated from L-arginine by the action of nitric oxide synthase (NOS)—or by the reaction of hydrogen peroxide with nitrite ions (NO_2_^−^).

Cells continually produce small, although highly reactive, ROS that originate from incomplete reduction of oxygen during aerobic metabolism. Most of the oxygen molecules undergo complete reduction to water, although partial reduction accompanied by ROS generation also occurs. Although ROS production may take place in the cytosol and plasma membrane through the activity of nicotinamide-adenine-dinucleotide phosphate (NADPH) oxidases (NOS) [93], it primarily occurs in mitochondria, the major source of ROS. Over ten different reported production sites have been described within the organelle [94], leading to the formation of superoxide anion radicals (O_2_**^•−^**), the most common species, as well as hydrogen peroxide (H_2_O_2_), and hydroxyl radicals (HO^•^) [95]. The most reactive superoxide anion is generated by electrons that leak from the electron transfer system located in the inner membrane of mitochondria. The process takes place particularly at the levels of complex I and complex III [90], although the exact mechanism and site of generation of these anions remains to be fully clarified (Figure 1). Indeed, there is great variability reported in the literature concerning the rate and substrate specificity of mitochondrial superoxide and H_2_O_2_ production, with sometimes controversial and difficult to compare results [90]. Reported differences in the site of ROS generation within the electron transport chain also relate to the tissue origin of the organelles, with complex III being an active source in the case of heart and lung mitochondria but exerting a more debatable role in the brain [90,96,97]. Increasing the complexity of the mechanisms involved, non-respiratory chain enzymes with ability to produce superoxide in mitochondria, such as glycerol-3-phosphate dehydrogenase, have also been reported, although their contribution to total mitochondrial ROS production remains unclear [98].

Under normal physiological conditions, the formation and elimination of ROS is highly controlled by endogenous antioxidant systems and ROS scavengers to avoid oxidative stress and toxicity. In this context, superoxide ions produced in the mitochondria have a very short half-life. They are typically dismutated into molecular oxygen and hydrogen peroxide by different superoxide dismutase isoforms that differ in the metal cofactor at their active site [99], such as Mn-SOD in the mitochondrial matrix or Cu/Zn-SOD in the intermembrane space and the cytosol [100,101,102,103,104]. In turn, hydrogen peroxide, which is also continuously produced endogenously through NADPH oxidase (NOX), mitochondrial oxidative phosphorylation, as well as by other non-mitochondrial cell compartments including peroxisomes and endoplasmic reticulum, is also a ROS with an unstable peroxide bond and the capacity to oxidize proteins, lipids, and DNA. Since, in addition to this detrimental potential on the cellular function, H_2_O_2_ can also act as a signaling molecule with capacity to regulate various biological processes including vascular remodeling [105,106], its homeostasis is a highly regulated process. The molecule is either normally converted to molecular oxygen and H_2_O by catalase, or reduced enzymatically by mitochondrial/cytosolic peroxiredoxins and glutathione peroxidases. These processes transfer the energy of the peroxide to either thioredoxin—a small protein with two crucial cysteines, Trx(SH)_2_—or to GSH, a small sulfur-containing tripeptide, respectively [88,107,108,109,110,111]. These oxidable thiols, known as sulfur or thiol switches, are crucial for controlling the cellular redox state and assuring the maintenance of protein structures and regulation of enzymatic function, as well as receptor, transcription factor, and transporter activities [112]. In fact, the ratio of GSH to GSSG is considered a marker of oxidative stress [113] and an imbalance in GSH homeostasis has been correlated with pro-oxidizing states linked to normal aging and further altered in association with synaptic dysfunction in neurodegenerative diseases including Parkinson’s and Alzheimer’s disease [88,114,115].

Hydroxyl radicals (HO^•^ and HOO^•^) are typically generated through the Fenton reaction from H_2_O_2_ in the presence of transition metals such as iron [88,109] (Figure 1). These extremely reactive and short-lived hydroxyl molecules, because of their high reactivity, cannot act as substrates for any enzyme, which precludes their removal by enzymatic reactions [116]. In fact, hydroxyl radicals are neutralized by interaction with adjacent oxidizable molecules, resulting in genomic instability [117], and leading to the formation of covalently crosslink proteins, and oxidation products of nucleic acids, amino acids, and lipid molecules [118,119]. In this sense, levels of the DNA oxidative damage biomarker 8-hydroxy-deoxyguanosine have been shown to be elevated in AD and animal models of the disease [120].Certainly, based on the particular abundance of lipids at cellular membranes, lipid peroxidation resulting from the attack of these free radicals on nucleophilic polyunsaturated fatty acids readily leads to the formation of lipid hydroperoxides such as malondialdehyde, 4-hydroxynonenal (HNE), 4-oxo-2-nonenal, and acrolein, thereby causing membrane lipid degradation and irreversible cell damage [109]. Indeed, HNE, which is often regarded as a marker of oxidative stress [121], is present in increased levels in AD plasma and is found to be associated with Aβ lesions in AD brains [118,122].

### 3.2. Production and Regulation of Reactive Nitrogen Species

Reactive nitrogen species (RNS) are constituted by various nitric oxide-derived compounds and include, among the most active radicals, nitric oxide and peroxynitrite [123,124]. While, in general, RNS play crucial roles in the physiologic regulation of many—if not all—living cells and possess pleiotropic properties on multiple cellular targets, their elevated levels have been implicated in cell injury and death, similarly to what it occurs with ROS. One of the most relevant nitrogen species is NO, a critical intracellular messenger that regulates essential physiological functions but becomes deleterious under pathological conditions as a result of its high reactivity with other free radicals, such as the superoxide anion, as described below. Nitric oxide synthase (NOS)—of which three different isoforms exist in mammalian cells—mediates the generation of NO through downstream processing of L-arginine (Figure 1). Despite their similar functions, the three NOS isoforms exhibit different expression profiles, with nNOS being constitutively expressed in neurons, iNOS in non-neuronal cells such as macrophages, astrocytes, and microglia, and eNOS mainly present in endothelium [125]. All three NOS subtypes consume L-arginine and molecular oxygen as substrates and utilize NADPH as the electron donor to produce the NO^•^ radicals that exhibit one free electron in the outer pi molecular orbital [126].

More reactive than NO^•^ species, which show an extremely short half-life in vivo and relatively short-distance coverage, are the highly active peroxynitrite radicals, ONOO^•^ [127]. These reactive forms result from the crosstalk between ROS and RNS paths and are generated by the rapid reaction between NO^•^ and O_2_^•−^ (Figure 1). Peroxynitrite radicals possess a strong oxidant activity that primarily nitrates tyrosine residues via non-enzymatic reactions, a post-translational modification that leads to protein misfolding and aggregation, ultimately disrupting protein activity [126,128]. Highlighting the relevance of RNS for disease pathogenesis, increased levels of nitrotyrosine-modified proteins, a marker of peroxynitrite-mediated oxidative stress, have been reported in AD brain tissues and increased nitration of manganese superoxide dismutase was described in cerebrospinal fluid from AD patients, as well from individuals with other neurodegenerative conditions [129].

The mechanisms that protect mammalian cells from the deleterious effects of RNS stress remain to be clarified in detail. Nonenzymatic protection is provided by diverse cellular antioxidant systems, including GSH, metalloporphyrins, β-carotenes, and vitamins E and C [123]. In addition, although not as thoroughly studied as in their role in ROS regulation, thioredoxin, GPx, SODs, and γ-glutamyl transpeptidase have also been recognized as potential systems for RNS elimination [123,130]. NO^•^ is rapidly removed by its rapid diffusion through tissues into red blood cells, where it is rapidly converted to nitrate by reaction with oxyhemoglobin [131,132]. In contrast, peroxynitrite is a strong oxidant with capacity to react directly with electron-rich groups, such as sulfhydryls, iron-sulfur centers, zinc-thiolates, and the active sulfhydryl sites in tyrosine phosphatases [127,133,134]. The radical can also interact with proteins that contain transition metal centers and therefore is able to modify proteins such as hemoglobin, myoglobin, and cytochrome c by oxidizing their respective ferrous heme groups into their corresponding ferric forms. The strong oxidative properties of peroxynitrite also have profound effects on the structure and function of proteins, through their ability to oxidize different amino acids in the peptide chain. The most commonly modified residues are cysteines, which incorporate nitrosyl radicals into select thiol groups through S-nitrosylation, a post-translational change capable of altering the catalytic activity of enzymes, modifying cytoskeletal organization, and causing impaired signal transduction [127,135]. As seen with other RNS-mediated protein modifications, S-nitrosylation, as a result of its structure modifying properties, is often associated with protein misfolding, as well as with the induction of apoptosis, mitochondrial alterations, and synaptic dysfunction, characteristics that are often associated with AD and other neurodegenerative disorders [136].

## 4. Mitochondrial and Bioenergetic Alterations in Alzheimer’s Disease

Mounting evidence indicates that mitochondrial function is a crucial element in the molecular mechanisms leading to AD pathophysiology. Alterations in mitochondrial dynamics, deficiencies in mitochondrial proteins, changes in mitochondrial membrane potential, and an increase in oxidative stress have been observed associated with the disease [47,48,49,52,70,85,86,87]). Among these abnormalities, numerous reports have demonstrated alterations in mitochondrial number and transport as well as changes in morphology, with altered shapes, fewer cristae, and variations in size, including the presence of both enlarged, very small, and elongated organelles [68,137,138]. Also present in AD is the reduced expression and activity of the electron transport chain (ETC) enzymes. AD mitochondria show a generalized depression in the activity of all transport complexes, with a more marked decrease in the case of the complex IV cytochrome c oxidase as well as in ATP synthase, alterations that compromise mitochondrial membrane potential and ATP production [86,138,139,140]. This dysregulation of mitochondrial pathways is triggered in vitro by oligomeric forms of Aβ [29,141,142,143,144,145,146] and has been recapitulated in transgenic animal models [53,54,55,56,57,58,59,60,61,62,63,64,65,66,67,68,69], which demonstrated not only increased generation of free radicals and oxidative DNA/protein damage but also reduced glucose metabolism/ATP production [43,147,148,149,150,151,152,153,154]. In cell culture models, it has been reported that oligomeric Aβ induces mitochondria-mediated changes in neuron metabolism, with a significant reduction in basal respiration levels evaluated in Seahorse platforms [51,155]. In these assays, an Aβ-mediated decrease in O_2_ consumption was also observed, associated with the generation of ATP as well as a diminished maximal respiration capacity measured through the addition of the proton ionophore carbonyl cyanide-4-(trifluorome-methoxy)phenylhydrazone (FCCP) [51,155]. This compound allows free proton permeability across the mitochondrial inner membrane, disrupting, in turn, ATP synthesis and stimulating the respiratory chain to operate at maximum capacity, only limited by substrate availability [156,157,158].

**Figure 2:**
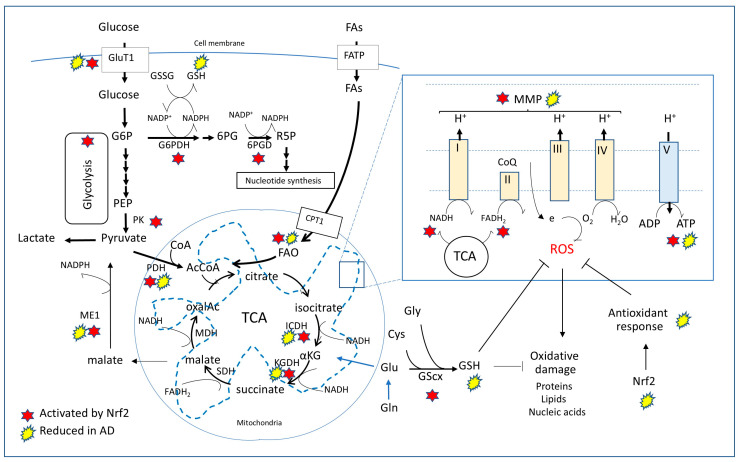
Role of Nrf2 in cellular metabolic/bioenergetic pathways. Nrf2 has the capability to activate and modulate multiple cellular components that are involved in energy production, among them the glucose receptor Glut1, enzymes associated with glycolysis, the pentose phosphate pathway, the TCA cycle, anaplerosis, and fatty acid oxidation, as well as elements regulating ATP production at the mitochondrial electron transport chain (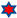 ) (red star symbol). Notably, many of these integral components, including the transcription factor Nrf2 itself with its concomitant downstream antioxidant response, have been reported downregulated in AD (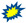) (yellow star symbol). Thus, enhancing Nrf2 expression and/or upregulation may constitute an attractive translational approach in the field of neurodegeneration and AD, with potential to exert a positive impact not only in the metabolic aspects of the disease but also in boosting the antioxidant response and reducing the detrimental effects of oxidative stress on proteins, lipids and nucleic acids. Complex V of the respiratory chain, also known as ATP synthase, is represented in light blue while Complexes I-IV are illustrated in color beige to differentiate their function. Complex V catalyzes the synthesis of ATP using the proton gradient generated by the complexes I-IV. Abbreviations: G6P, Glucose 6 phosphate; PEP, phosphoenolpyruvate; PK, pyruvate kinase; ME1, malic enzyme 1; PDH, pyruvate dehydrogenase; ICDH, isocitrate dehydrogenase; αKG, alpha ketoglutarate; KGDH, ketoglutarate dehydrogenase; SDH, succinate dehydrogenase; OxalAc, oxaloacetate; CS, citrate synthase; FAs, fatty acids; FATP, fatty acid transport proteins; FAO, fatty acid oxidation; GScx, glutathione synthase complex; MMP, mitochondrial membrane potential; TCA; tricarboxylic acid; CoQ; coenzyme Q; AcCoA; acetyl-coenzyme A; MDH; malate dehydrogenase 6PGD; 6-phosphogluconate dehydrogenase; G6PDH; glucose-6-phosphate dehydrogenase; R5P; ribose 5-phosphate; CPT1, carnitine palmitoyltransferase 1.

Cerebral glucose hypometabolism is a pathophysiological feature recognized as a critical contributor to AD [159,160]. Impairment of glucose metabolism, particularly in synapse rich areas, was demonstrated by ^18^FDG-PET (^18^fluorodeoxyglucose-positron emission tomography) [161,162,163,164] and is a feature that precedes the onset of the histopathological hallmarks and symptoms [139,165,166]. These studies have shown a low rate of glucose metabolism—with levels about 20 to 30% lower than those found in healthy individuals—in brain regions involved in processing memory, including the hippocampus as well as posterior cingulate, temporal, and parietal cortex [167]. ^18^FDG-PET is based on the capture of the radioactive glucose homologue ^18^FDG by metabolic active cells. It is an earlier indicator of hypometabolism associated with neuronal dysfunction, but also a useful marker of neuronal loss and brain atrophy in later stages of the disease [168,169]. The combination of structural MRI with ^18^FDG-PET allows the simultaneous evaluation of changes in brain volume (atrophy) and brain metabolic activity (hypometabolism) [166,170]. Since neuronal dysfunction precedes neuronal loss and brain atrophy, ^18^FDG-PET is a useful tool to identify individuals at risk for AD prior to the onset of cognitive symptoms [162,170,171]. Supporting these metabolic abnormalities, biochemical analyses have shown, in correlation with cognitive impairment, alterations in the activity of key enzymes participating in the tricarboxylic acid (TCA) cycle—pyruvate dehydrogenase (PDH), α-ketoglutarate dehydrogenase (KGDH), and isocitrate dehydrogenase (ICDH)—together with changes in the activity in malic enzyme (ME1), an important anaplerotic element in neurons (Figure 2) [172,173,174,175,176,177,178,179,180,181]. Indeed, a comprehensive longitudinal study indicated overall brain glucose dysregulation starting several years before the onset of AD, showing impaired glucose transport, reduced glycolytic flux, and decreased activity of glycolytic enzymes coexisting with elevated glucose brain tissue levels [182]. This increased brain glucose concentration, also observed by magnetic resonance spectroscopy in AD patients subjected to glucose overload, is consistent with the hypometabolism showed by ^18^FDG-PET [183], supporting the notion that glucose accumulation in certain brain areas may occur concomitantly with a decreased ability of the organ to metabolize the compound. Although estimations of human brain glucose metabolism by ^18^FDG-PET are very sophisticated in measuring regional glucose utilization, they are limited in their capacity to identify the underlying mechanisms. In this sense, and adding to the complexity of brain glucose metabolism, contrasting early biochemical studies on cortical biopsies demonstrated increased glucose oxidation and partial oxidative phosphorylation uncoupling in the respiratory chain [17]. This mitochondrial uncoupling with proton leaks across the inner organelle membrane and decreased ATP production may, in turn, lead to a complex interplay with other factors triggering compensatory mechanisms for energy production, including increased glucose uptake and a shift to glycolytic metabolism [184].

## 5. Mitochondrial and Bioenergetic Alterations in Transgenic Models

Highlighting mitochondrial alterations in APP transgenic models, hippocampal synapsis exhibited early mitochondria-associated alterations [79], including an increase in Aβ within synaptic mitochondria, leading to the organelle dysfunction and oxidative stress prior to plaque accumulation [68]. Furthermore, proteomic and metabolomic analyses in different mouse models, including Tg2576, APP/PS1, and 3xTg, showed—in addition to oxidative stress—an early dysregulation in mitochondrial and synaptosomal proteins [185,186]. These alterations occurred in conjunction with impaired amino acid metabolism, a compensatory increase in the fatty acid beta-oxidation (FAO) pathway, which is a major mechanism producing energy from fats through the breakdown of fatty acids into acetyl-CoA, and an overall decrease in energy metabolism (Figure 2) [186,187]. Supporting the significance of impaired brain glucose metabolism/uptake for AD pathophysiology, it has been reported that APP/PS1 mice exhibiting a decreased glucose uptake caused by experimental reduction of endothelial GLUT1 transporter—a protein responsible for the facilitated diffusion of glucose across plasma membranes—showed dramatically exacerbated cognitive defects [188]. Overall, the increasing evidence linking bioenergetic changes with AD pathobiology suggests that targeting early synaptic deficits by preventing the effects of Aβ on mitochondrial dysfunction constitutes a potentially effective avenue to prevent cognitive loss [189].

## 6. Linking ROS Homeostasis and Mitochondria-Associated Metabolic Pathways Through Nrf2

Despite extensive research, the links among ROS homeostasis, mitochondria bioenergetics, and metabolic stress, particularly at the level of synapsis and the microvasculature and in relationship with oligomeric Aβ, remain not completely elucidated. A central element interlinking these complex pathways is Nrf2 (nuclear factor erythroid 2-related factor 2). This is a crucial transcription factor that regulates the expression of over 500 cytoprotective genes [190,191,192], modulating inducible defense systems [193] and regulating not only the antioxidant response [190,191] but also mitochondria functioning, as well as multiple points of the cell intermediary metabolism [191]. Nrf2 is a cytosolic protein with constitutive low levels that are controlled by the proteasome [194]. Under certain pathological and stress conditions, Nrf2 degradation is severely reduced. This leads to cytoplasmic accumulation of the transcription factor and subsequent translocation to the nucleus, where Nrf2 can bind the antioxidant response elements (AREs) motifs found in the promoter region of several genes encoding detoxification and cytoprotective proteins, among them NAD(P)H-quinone oxidoreductase (NQO1), glutathione-S-transferase (GST), heme-oxygenase 1 (HO-1), and SOD, initiating the transcription and protein expression of the antioxidant genes [195] in a process known as the antioxidant response.

### 6.1. Structural Characteristics of Nrf2

**Figure 3:**
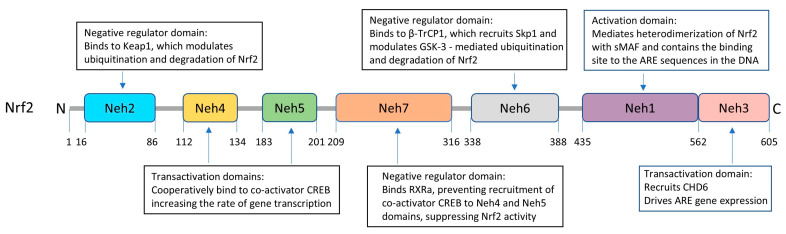
Structural characteristics of Nrf2. The transcription factor Nrf2 is a 605 amino acid long modular protein comprising seven highly conserved regions, Nrf2-ECH homology (Neh) domains. These domains have distinct functional activities, extending from those responsible for the binding to Keap1 or β-TrCP that mediate Nrf2 ubiquitination and degradation to those a driving the binding to the ARE motifs in the DNA and the gene expression of enzymes associated with the antioxidant response. Abbreviations: Keap1, Kelch-like ECH-associated protein 1; β-TrCP1, β-transducing repeat-containing protein 1; CREB, cAMP response element-binding protein; RXRa, retinoid X receptor; Skp1, S-phase kinase-associated protein 1; sMAF, small musculoaponeurotic fibrosarcoma protein; ARE, antioxidant response element; GSK-3; glycogen synthase kinase 3.

Human Nrf2 is a 605 amino acids-long protein that belongs to the family of basic leucine zipper (bZIP) transcription factors [196]. Nrf2 is a modular protein comprising seven highly conserved regions, known as Nrf2-ECH homology (Neh) domains (Figure 3). Neh1 contains the highly conserved CNC-bZIP region that mediates heterodimerization with the small musculoaponeurotic fibrosarcoma (sMAF) proteins. This domain is essential for Nrf2 binding to its target ARE motif, a cis-acting enhancer sequence present in the promoter region of many genes encoding antioxidant and detoxification proteins [191,197,198]. The Neh2 region negatively controls the activity of Nrf2; it contains two highly conserved peptide sequences, DLG and ETGE, capable of binding Kelch-like ECH-associated protein 1 (Keap1) which mediates the ubiquitination and degradation of Nrf2 [199,200]. The carboxy-terminus Neh3 region is a transactivation domain that recruits the chromo-ATPase/helicase DNA-binding protein 6 (CHD 6) and drives ARE gene expression. Both Neh 4 and Neh 5 are also transactivation domains acting cooperatively in binding the co-activator cAMP response element-binding protein (CREB), thereby synergistically increasing the rate of gene transcription [201]. Meanwhile, Neh6 domain is a serine-rich region that negatively regulates Nrf2 stability and is responsible for the Keap1-independent regulation of Nrf2 [202] (Figure 3). The domain contains two highly conserved peptide motifs, DSGIS and DSAPGS, that are recognized by β-TrCP (β-transducing repeat-containing protein) which, in turn, serves as a substrate receptor for the S-phase kinase-associated protein 1- Cullin 1- RING box protein-1/regulator of cullins-1 (Skp1–Cul1–Rbx1/Roc1) ubiquitin ligase complex [198,200]. Neh7, the most recently described Nrf2 domain, can engage in a direct protein–protein interaction with the DNA-binding domain of retinoid X receptor a (RXRa), causing suppression of Nrf2 activity by preventing recruitment of coactivators to the Neh4 and Neh5 domains [203].

### 6.2. Regulation and Activation of Nrf2

**Figure 4:**
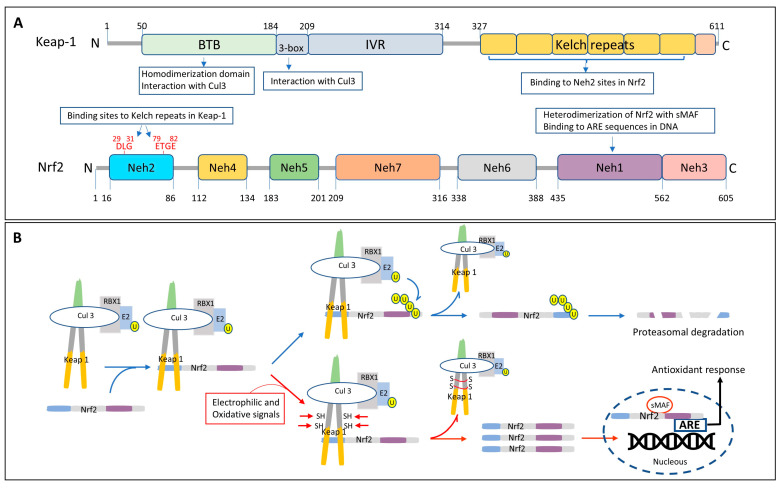
Interaction of Nrf2 with Keap1. (**A**) Domain organization of Keap1 and Nrf2, highlighting the regions directly involved in their interaction. The binding sites on the Neh2 domain of Nrf2 to the Kelch repeats in Keap1 are indicated in red font. (**B**) Schematic representation of the Keap1-E3 ubiquitin ligase complex and its interaction with Nrf2. Keap1, as part of the E3 ubiquitin ligase complex together with Cullin 3 and RBX1, is able—in normal conditions—to interact with Neh2 domain of Nrf2, allowing its ubiquitination and subsequent proteasomal degradation (blue arrows). In the presence of electrophilic compounds and/or oxidative signals, the oxidation of specific sulfhydryl residues in the Keap1 (red arrows) induces the release of Nrf2 form the Keap1-E3 ubiquitin ligase complex resulting in an increase of cytoplasmic Nrf2, subsequent translocation to the nucleus, binding to the ARE sequence motif in the DNA, and initiation of the antioxidant response. (U) represents ubiquitin molecules. Abbreviations: Keap1, Kelch-like ECH-associated protein 1; BTB, Broad complex-Trantrack-Bric à Brac; IVR, intervening region; Neh, Nrf2-ECH homology; Cul3, Cullin 3; RBX1, Ring box 1; E2, E2 ubiquitin ligase; U, ubiquitin; E3 ubiquitin ligase complex, Cul3-RBX1, where the E2-ubiquitin conjugate binds; sMAF, small musculoaponeurotic fibrosarcoma protein; ARE, antioxidant response element.

Different mechanisms have been described for the activation of Nrf2, the most studied of which is the one involving the Keap1 pathway. Keap1, through its Kelch repeats and as part of the E3 ubiquitin ligase complex in conjunction with Cullin 3 and Ring-Box1 (RBX1), is capable of interacting with the Neh2 domain of Nrf2 at specific motifs (residues 29–31 and 79–82) (Figure 4A), allowing its ubiquitination and subsequent proteasomal degradation, thereby maintaining the low endogenous levels of Nrf2 [196,204] (Figure 4B). Electrophilic molecules and/or oxidative signals are capable of modifying specific Cys residues in Keap 1, inducing the release of Nrf2 from the complex and preventing its consequent proteasomal degradation. As a result, there is an increase in cytoplasmic Nrf2 that is followed by its nuclear translocation, its association with the Small Musculoaponeurotic Fibrosarcoma protein (sMAF) and its binding to the ARE sequence motif in the DNA, with the subsequent initiation of the antioxidant response [196,205,206] (Figure 4B).

**Figure 5:**
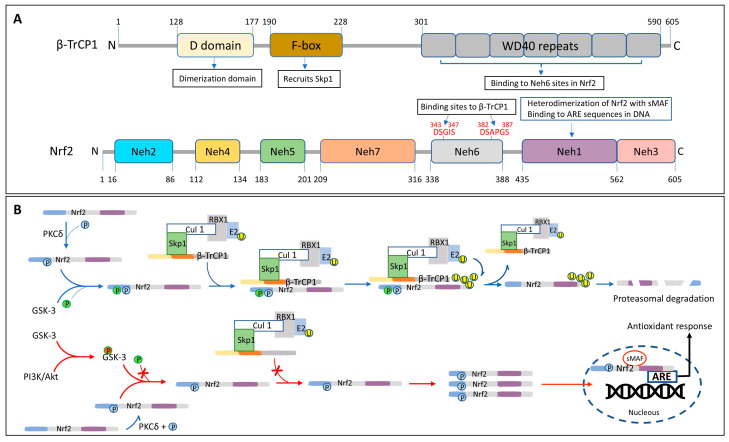
Interaction of Nrf2 with β-TrCP1. (**A**) Domain organization of β-TrCP1 and Nrf2, highlighting the regions directly involved in their interaction. The binding sites on the Neh6 domain of Nrf2 to the WD40 repeats in β-TrCP1 are indicated in red font. (**B**) Schematic representation of Nrf2 interaction with the β-TrCP1-E3 ubiquitin ligase complex, which requires previous phosphorylation of the transcription factor by PKCδ and GSK-3. Once phosphorylated, Nrf2 binds to the β-TrCP1-SCF ubiquitin ligase complex assembled with Skp1, Cul1, and RBX1—where the E2-ubiquitin conjugate binds—allowing ubiquitination of Nrf2 and degradation by the proteasome (blue arrows). Previous GSK-3 phosphorylation by PI3k/Akt renders GSK-3 unable to phosphorylate Nrf2, preventing its interaction with the β-TrCP1-SCF ubiquitin ligase complex and precluding its subsequent proteasomal degradation (red arrows). As a result, Nrf2 accumulates in the cytosol and it is translocated to the nucleus where it binds to the ARE motif, initiating the transcription of target genes and the subsequent antioxidant response. (U) represents ubiquitin molecules. (P) illustrate phosphate molecules added by PKCδ (light blue), GSK-3 (green), and PI3k/Akt (red). Abbreviations: β-TrCP1, β-transducing repeat containing protein 1; D domain, dimerization domain; Neh, Nrf2-ECH homology; PKCδ, protein kinase C-δ; GSK-3, glycogen synthase kinase-3; Cul1, Cullin 1; Skp1, S-phase kinase associated protein 1; RBX1, Ring box 1; sMAF, small musculoaponeurotic fibrosarcoma protein; ARE, antioxidant response element.

Although Keap1 is the most studied regulator of Nrf2, a different E3-ubiquitin ligase adaptor molecule, β-TrCP, has also been described [191]. This mechanism, independent of Keap1, involves the interaction of the WD40 repeats in β-TrCP with specific Nrf2 binding motifs located in the Neh6 domain of Nrf2 (Figure 5A). The process is regulated by glycogen synthase kinase 3 (GSK-3), which phosphorylates the DSGIS motif of Nrf2, promoting the recruitment of the multi-protein ubiquitin ligase complex formed by β-TrCP together with the Skp1 adaptor, Cullin 1, and Rbx1, targeting the molecule for proteasomal degradation [207,208,209] (Figure 5B). The PI3K/Akt pathway, through phosphorylation and inactivation of GSK-3, renders GSK-3 unable to phosphorylate Nrf2, preventing its interaction with the β-TrCP1 complex and precluding its ubiquitination and degradation. As a result, Nrf2 accumulates in the cytosol and it is translocated to the nucleus where it binds to the ARE motif, initiating the transcription of target genes and the subsequent antioxidant response [191] (Figure 5B).

### 6.3. Nrf2 Network Links Cell Metabolic Paths, Redox Homeostasis, and Blood-Brain Barrier Integrity

In recent years, additional functions of Nrf2 have been discovered that go beyond the classical view of Nrf2 as a mere master regulator of antioxidant responses and indicate its complex/multifaceted activity and its critical role in the modulation of cellular bioenergetics, metabolic regulation, and response to nutrient shifts. In this sense, Nrf2 has been shown to regulate mitochondrial bioenergetics and its loss translates to decreased mitochondrial membrane potential, reduced ATP production, and altered cellular respiration [210]. The crucial role of Nrf2 in cell bioenergetics results in part from its capacity to regulate the biosynthesis of key molecules responsible for the maintenance of the redox homoeostasis, including thioredoxin and glutathione. Accordingly, both the catalytic and the regulatory subunits of γ-glutamyl cysteine ligase (GCLC), the enzyme that catalyzes glutathione biosynthesis, are encoded by genes regulated by Nrf2 [211]. Nrf2 is also involved in the generation of NADPH, a crucial component for the maintenance of ROS homeostasis, by regulating the gene expression of glucose-6-phosphate dehydrogenase (G6PD), pyruvate dehydrogenase (PDH), isocitrate dehydrogenase (ICDH-1), ketoglutarate dehydrogenase (KGDH), and malic enzyme-1 (ME-1) (Figure 2) [212,213,214,215]. As a result of its ability to modulate all these enzymatic pathways, Nrf2 is capable of influencing glycolysis, TCA cycle activity, substrate availability for respiration, efficiency of oxidative phosphorylation, and ATP production [190,210], overall regulating cellular metabolic shifts [191,213,214,215].

Adding to these wide-range of metabolic roles, Nrf2 is intricately connected to the control of lipid metabolism as well as to the regulation of mitochondrial fatty acid oxidation, which provides up to 90% of the total fatty acid-derived energy [216,217]. Accordingly, it has been shown that both mitochondria-mediated oxidation of long- and short-chain fatty acids, as well as the rate of FADH2 regeneration—which is produced during the α-β dehydrogenation of the acyl-CoA fatty acid ester—are depressed in the absence of Nrf2 and accelerated when Nrf2 is constitutively active [217]. In addition, Nrf2 plays an important role in lipid metabolism. Its capacity to negatively modulate lipid biosynthesis was demonstrated by the downregulation of ATP-citrate lyase, fatty acid synthase, and stearoyl CoA desaturase, three critical enzymes involved in fatty acid synthesis, under conditions of Nrf2 activation in Keap1-KO models or following pharmacological activation of the transcription factor [218]. Overall, Nrf2 has a significant impact on the efficiency of fatty acid oxidation, ultimately affecting mitochondrial metabolism and bioenergetics, properties that suggest a contribution of this metabolic role of Nrf2 in the pathophysiology of certain chronic disease conditions, including cancer and neurodegeneration.

The multiphasic activity of Nrf2 extends to its ability to control endothelial cells’ (EC) homeostasis and overall BBB function. Multiple lines of investigation have demonstrated that Nrf2-mediated antioxidant pathways exert important physiological roles for vascular protection in aging and age-associated conditions, as covered in detail in comprehensive reviews [219,220,221,222]. Among the most relevant target genes associated with the protective Nrf2 pathway is the antioxidant defense protein HO-1, one of the most important ARE-driven antioxidant enzymes in endothelial cells, which, together with NQO1, GST, and Trx, has been shown to effectively prevent EC dysfunction [221,223]. Another antioxidant response regulated by Nrf2 is the transcription of the catalytic subunit of GCLC, a rate-limiting enzyme that regulates GSH biosynthesis [224]. This tripeptide (cysteine, glycine, and glutamic acid) is a major endogenous antioxidant that has been shown to exert a relevant role in the maintenance of EC function, preventing ROS-mediated apoptosis [225].

In addition of protecting vascular EC from oxidative stress through the canonical activation of its target genes, Nrf2 has been shown to counteract BBB disruption by regulating inflammatory processes associated with endothelial dysfunction, as well as by preserving tight junction integrity and preventing overall barrier membrane leakage [221]. Accordingly, in vitro and in vivo models have demonstrated the ability of Nrf2 to regulate the expression of the tight junction proteins Occludin and Claudin-5 as well as that of the adherens junction protein VE-Cadherin, crucial molecules for the regulation of BBB transport and barrier strength [220,226]. An important additional element to consider in the maintenance of BBB integrity is the activity of enzymes participating in the physiological extracellular matrix remodeling, in particular matrix metalloproteases (MMPs). In this context, it is known that the MMP-2 and MMP-9 isoforms of the enzymes are present in the extracellular matrix surrounding the brain endothelium [227]. MMP-9 is particularly harmful to BBB integrity, as it can hydrolyze not only the extracellular matrix but also tight junction proteins, contributing in this way to BBB leakage and microhemorrhages [220,227,228]. Notably, the activities of MMPs, in addition to being tightly regulated by specific tissue inhibitors, can be modulated through Nrf2 signaling, as demonstrated by the elevated expression of the enzymes in Nrf2 knockout mice [229]. The relevance of the transcription factor for EC function has been further highlighted by the recent generation of an endothelial cell-specific Nrf2 knockout model that resulted in an impaired brain EC homeostasis and overall reduced EC barrier strength [219].

### 6.4. Nrf2 in Aging and Alzheimer’s Disease

Studies on the central nervous system (CNS) of both humans and mice demonstrated that Nrf2 is expressed in neurons, astrocytes, and glial cells, with astrocytes exhibiting higher levels than neurons [230,231]. A decline in Nrf2 expression and activity that impacts its target genes has been observed in aging and AD [232,233,234,235,236], with reports of lower expression of the transcription factor in association with increased risks and early onset of the disease [237]. Additionally, an impaired nuclear translocation of the transcription factor in association with AD is supported by the decreased levels of nuclear Nrf2 in neurons of the CA1 hippocampal region that take place despite the presence of oxidative stress markers, suggestive of potential mechanisms blocking Nrf2 nuclear activity as a contributor to neuronal dysfunction [233].

The observed deficits of Nrf2 expression in AD brains have been recapitulated in different AD animal models [238,239] and the genetic deletion of Nrf2 further exacerbated the cognitive deficits in spatial learning and memory observed in APP/PS1 mice [240]. In a different model, the APP knock-in mice, the induction of Nrf2 ameliorated cognitive impairment, suppressing oxidative stress and decreasing neuroinflammation [241]). The link between Nrf2 activation and restoration of mitochondrial metabolism is supported by research demonstrating that canonical Nrf2 activators such as sulforaphane (SFN) and other natural antioxidants, in addition to preventing Aβ-mediated apoptotic and oxidative mechanisms, also protect from amyloid-induced alterations in mitochondrial function, both in in vitro neuronal cell cultures and in vivo in transgenic mice [242,243,244,245,246,247]. Providing insight into the mechanisms involved in disease pathogenesis, in vitro cell culture studies demonstrated that Nrf2 activation through specific inhibition of Keap1 prevented Aβ-mediated neuronal toxicity and ROS generation [248]. In support of the relevance of the β-TrCP path in Nrf2 modulation in AD, it is noteworthy to mention that an increased activity of GSK-3 has been shown in patients with the disease, consistent with the decreased activation of Nrf2 observed in these cases [249,250,251,252].

Overall, all the above-mentioned findings in diseased individuals and animal models strengthen the link between Nrf2 and neurodegeneration suggesting the modulation of the transcription factor as an important potential therapeutic target for AD; this will be considered in detail below, along with the action of other protective compounds.

## 7. Therapeutic Strategies for Alzheimer’s Disease Targeting Oxidative Stress and Mitochondrial Dysfunction

The complex and progressive pathological phenotype of AD supports the concept that successful treatment strategies will require multifaceted and disease stage specific approaches. The research described above, sustaining a crucial and causal role of mitochondria in bioenergetic deficits, brain hypometabolism, and dysregulation of ROS homeostasis, indicates that targeting different aspects of mitochondria-mediated paths is likely to contribute to the development of effective therapeutic strategies. Indeed, several promising compounds modulating these pathways are currently under preclinical or clinical evaluation as alternative or complementary therapeutic strategies in either mild cognitive impairment or AD. Extensive reviews are available in the literature, addressing in-depth current antioxidant therapeutic approaches [253,254,255,256,257,258]; therefore, we will limit this section to the strategies more closely related to the cellular pathways covered in this article.

### 7.1. Mitochondria-Targeted Antioxidants

Candidate compounds able to potentiate mitochondrial bioenergetics and enhance brain glucose metabolism are expected to promote healthy aging and counterbalance the brain hypometabolism preceding AD clinical manifestations, ultimately preventing/ameliorating the disease. This category of compounds comprises multiple naturally occurring herbals and co-factors, among them R-α-lipoic acid and coenzyme Q10 [259]. Lipoic acid, with a potent antioxidant capacity, is capable of upregulating mitochondrial bioenergetics, promoting glucose uptake and metabolism, and suppressing oxidative stress [260]. The naturally occurring fatty acid is an important co-factor for the key mitochondrial enzymes PDH and KGDH, which—as illustrated in Figure 2—are also modulated by Nrf2. Highlighting its therapeutic potential, chronic administration of lipoic acid—although not altering Aβ levels or plaque deposition—was shown to decrease lipid peroxidation markers and improve hippocampal-dependent memory deficits in Tg2576 mice [261,262]. Coenzyme Q10 is a potent antioxidant and an essential component of the electron transport chain that accepts electrons from complex I and II [263]. Various studies have shown decreased levels of coenzyme Q10 in patients with neurodegenerative conditions [264,265] and recent findings seem to indicate an association of low levels of the coenzyme with the risk of dementia [266]. Underscoring its potential therapeutic capability, supplementation with coenzyme Q10 has been reported to protect neuronal cell cultures from oxidative damage, improve learning in aged mice, reduce neuronal degeneration in transgenic models of different neurodegenerative conditions including Huntington’s, Parkinson’s, and Alzheimer’s diseases, and ameliorate behavioral deficits in transgenic AD mice [267,268,269].

### 7.2. Phytochemicals and Natural Antioxidants

Several natural antioxidants from fruits, grains, and other plant foods are likely to offer therapeutic benefits resulting from their known ability to counterbalance free radicals. Minerals, vitamins, carotenoids, flavonoids, and phenolic acids are some of the most commonly studied compounds in this category [270,271]. Among the minerals with known antioxidant activity, selenium is one of the most widely studied [253,272]. Its importance in brain homeostasis results from its role in mitochondrial dynamics, regulation of calcium channels, and maintenance of redox balance. Glutathione peroxidase and thioredoxin reductase are selenium-containing enzymes featuring selenocysteine in their active sites that are essential for their catalytic activity (Figure 1). Interestingly, selenium has been recently identified as a direct electron donor to reduce ubiquinone, preventing lipid peroxidation and suppressing ferroptosis [273,274,275,276]. Evidence in humans indicates a negative association between selenium plasma levels and risk of cognitive decline in older adults [277]. In spite of these findings, studies aiming to use selenium as a therapeutic strategy for AD are limited and have provided controversial results, suggesting the need for additional research to fully elucidate the potential of the metal for prevention and/or treatment [273].

Vitamin E (α-tocopherol), vitamin C, and β-carotene have been shown to decrease free radical mediated damage in neuronal cells and help to ameliorate dementia pathogenesis [253,254]. Vitamin E, as well as its analogue Trolox, have been demonstrated to attenuate the detrimental effects of Aβ and to improve cognitive performance in rodents [278,279]. In AD mice, the decrease of oxidative stress induced by vitamin E supplementation correlated with reduced learning and memory deficits, concomitant with a decrease in Aβ deposition [254,280,281,282]. Although several randomized trials have investigated the efficacy of vitamin E as a potential therapeutic intervention for AD, the clinical benefits remain inconsistent and inconclusive [283]. This is also the case for vitamin C (ascorbic acid), the most extensively consumed antioxidant supplement [284]. Vitamin C deficiency has been associated with impaired cognition in aging mice and worsened Aβ deposition, oxidative stress, and cognitive abnormalities in the APP/PS1 Alzheimer’s mouse model [254,285]. In spite of this evidence, many studies of nutrition and cognition during healthy and abnormal aging in humans have not been as conclusive in supporting the role of this antioxidant to slow cognitive deterioration [284,286,287,288,289]. Translational discrepancies are not uncommon among studies investigating the role of oxidative stress in disease pathogenesis and potential therapeutic approaches [290,291]. Whether the discrepancies reflect weaknesses in the studies, limitations imposed by the selection of the biomarkers evaluated and the endpoints assessed, or are the result of a simplistic assessment of the role of oxidative stress in disease pathogenesis remains unclear.

One of the most promising phytochemicals with antioxidant activities is resveratrol, trans-3,4′,5-trihydroxystibene. This polyphenol, abundant in red grapes, blueberries, pomegranates, peanuts, and dark chocolate, exhibits diverse biological functions including, among others, modulation of glucose metabolism and inflammation-associated paths, as well as regulation of the activity of different enzymes therefore influencing multiple signaling mechanisms and cellular processes [254,271,292]. Resveratrol was shown to activate the transcription factor Nrf2, acting as a potent antioxidant in neurodegenerative disorders and treatment, with the compound causing significant reduction in Aβ deposition and improved cognition in different Tg models [293,294,295,296,297,298]. Consistent with these observations, moderate red wine consumption in APP Tg mice beneficially modulated AD-type deterioration and attenuated Aβ neuropathology [299]. In spite of the benefits provided by resveratrol treatment in animal models, its ability to scavenge ROS in vivo is hampered by its low bioavailability, as the chemical—like other dietary polyphenols including curcumin—has a short biological half-life, is quickly metabolized upon entering the body, and is rapidly eliminated [271,296,300]. A growing body of literature has focused on nanotechnology-based delivery systems for improving polyphenol bioavailability, including the use of liposomes, nanoparticles, and phospholipid complexes [301,302,303]. Whether these novel delivery strategies will help overcome the limitations affecting therapeutic potential of polyphenols in neurodegenerative conditions remains to be fully elucidated.

### 7.3. Compounds Targeting Nrf2 Pathway

Nrf2-mediated pathways—amply detailed above—play crucial roles in protecting the brain against ROS-mediated neurodegeneration, leading to the search for and development of numerous Nrf2 activators as potential therapeutic agents. Compounds that regulate Nrf2 include different chemicals, drugs, and natural herbal products [304]. The field is quite complex, since for some of the Nrf2 activators, the exact mechanisms underlying their protective action are not fully understood, whereas in the case of other compounds, their preventive effects take place through the engagement of multiple interlinking mechanisms of the broad Nrf2 pathway. It should also be mentioned that, despite their potential for the design of novel therapeutic strategies, some caution is required. In this sense, it has been shown that increased Nrf2 activity was associated with some types of cancer, cautioning against the prolonged use of Nrf2 activators without further investigations [305,306,307]. Some of the most common Nrf2 activators and their postulated mechanisms of action are described below. 

#### 7.3.1. Targeting Keap-l Mediated Nrf2 Degradation

**Figure 6:**
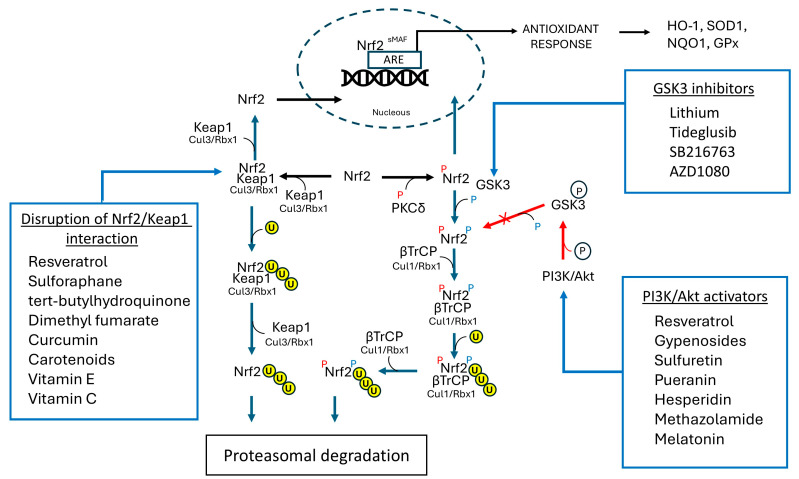
Nrf2 activators. Target location of various small molecule Nrf2 activators preventing the Keap1 and β-TrCP1 paths from directing Nrf2 for proteasomal degradation. These activators include (a) electrophilic compounds that disrupt the Nrf2-Keap1 interaction, (b) GSK-3 inhibitors that interfere with the GSK-3 phosphorylation of Nrf2 and prevent its interaction with the β-TrCP1, and (c) PI3k/Akt activators that allow the phosphorylation of GSK-3 and preclude further phosphorylation of Nrf2 and its subsequent interaction with β-TrCP1. (U) represents ubiquitin molecules. (P) illustrate phosphate molecules added by PKCδ (red), GSK-3 (light blue), and PI3k/Akt (black).

Several drugs target the release of Nrf2 from Keap-l. This category comprises electrophilic compounds which, upon acting on select cysteine residues on Keap-1, facilitate its release from Nrf2, resulting in the cytoplasmic accumulation of the transcription factor, subsequent nuclear translocation, and transcription of downstream protein targets (Figure 4B) [308]. Among the most studied compounds in this category are sulforaphane (SFN), tert-butylhydroquinone (tBHQ), curcumin, and dimethyl fumarate (DMF) (Figure 6) [236].

Sulforaphane (SFN) is an isothiocyanate found in cruciferous plants of the Brassicaceae family, such as broccoli, cabbage, and cauliflower, which activates Nrf2 by interacting with Keap1 cysteine thiols and has been proven to exhibit neuroprotective potential [304,309,310]. Various studies have shown that SFN, through Nrf2 pathway activation, increases the transcriptional expression of HO-1, NQO1, and other antioxidant enzymes, attenuating oxidative stress and cognitive dysfunction in different rodent models of vascular cognitive impairment and dementia, stroke, and brain injury [196,242,304,309,310]. SFN exerted also a protective effect on BBB leakage, upregulating the tight junction proteins occludin and claudin-5, along with Nrf2 expression [304]. In the context of AD, the compound was reported to prevent Aβ-mediated cell death as well as alterations in mitochondrial respiration and ATP generation in in vitro and in vivo models [242,245,310].

Tert-butylhydroquinone (tBHQ) is a synthetic phenolic antioxidant that is widely used as a food additive due to its low toxicity and low dosage requirement [311]. It has been shown to reduce oxidative stress and exert neuroprotection through Nrf2 activation in a number of neurodegenerative conditions, including brain damage resulting from cerebral ischemia [312,313,314]. In the AD field, studies in NT2N neurons, which express high levels of APP and generate intracellular Aβ, demonstrated that tBHQ treatment suppressed oxidative stress and concomitant caspase-mediated cell death [315]. Feeding APPPS1 transgenic mice with a tBHQ-enriched diet exerted multifactorial benefits. It increased the brain antioxidant capacity, elevating the concentration of glutathione and suppressing the expression of NADPH oxidase 2 with concomitant reduction in lipid peroxidation. In this mouse model, treatment with the phenolic antioxidant also stimulated Aβ enzymatic degradation pathways and modulated the expression of the low-density lipoprotein related protein-1, a multi-ligand endocytic receptor involved in clearing Aβ from the brain, collectively reducing brain Aβ load and supporting a more complex beneficial role of tBHQ than its mere antioxidant action [316].

Curcumin is a polyphenol from turmeric herbs that also exhibits antioxidant properties, both directly through scavenging free radicals and indirectly through upregulating the cytoprotective response [317,318,319]. Curcumin is capable of directly scavenging free radicals, reducing ferric ions, and chelating ferrous ions [317,318]. In addition, the compound was shown to upregulate the expression of genes encoding for antioxidant proteins, including HO-1, SOD, and catalase, while increasing the increase transcription of glutathione reductase that replenishes the antioxidant glutathione [319]. Studies in neuronal cell lines showed that the beneficial effects of curcumin treatment not only rescued glutathione and glutathione peroxidase levels, as demonstrated in non-neuronal counterparts, but also decreased oxidative stress-related detrimental changes, reducing the levels of lipid peroxidation and decreasing the expression of the pro-apoptotic caspase-3 and caspase-9 [317,320]. Using different AD mouse models, several investigators reported the in vivo beneficial effects of curcumin, which ameliorated cognitive decline and improved synaptic functions in different mouse models of AD [321]. The administration of the compound in APP/PS1 Tg mice enhanced spatial learning and memory, improving the quantity and structure of the synapses as well as the expression levels of synapse-related proteins [322,323,324]. These behavioral changes correlated with reduced hippocampal levels of Aβ and a concomitant increase in Aβ-degrading enzymes, suggesting a broad-spectrum beneficial action of curcumin [321,322]. Despite these benefits, the usefulness of curcumin as a therapeutic agent has been challenged by its low bioavailability, a common occurrence with other dietary polyphenols discussed above.

A major clinical success story among compounds activating Nrf2 through the electrophilic reaction with Keap1 thiol groups is dimethyl fumarate (DMF). A synthetic DMF analog has been approved by both the US Food and Drug Administration and the European Medicines Agency for the treatment of relapsing multiple sclerosis. This is a chronic inflammatory and degenerative disorder of the CNS in which oxidative stress has been shown as an important contributor to pathogenesis, particularly in the progressive forms of the disease [305,325,326,327,328]. In spite of numerous studies demonstrating an array of beneficial effects of the compound, DMF cellular and molecular targets remain not completely understood and may include both direct inhibition of proinflammatory pathways as well as activation of the Nrf2 antioxidant response [329,330]. Accordingly, the administration of DMF to a mouse model of multiple sclerosis increased Nrf2 expression in the nervous system and resulted in disease improvement, an effect not observed when treating mice lacking Nrf2 [305,326].

#### 7.3.2. Targeting TrCP-Mediated Nrf2 Degradation

A different therapeutic strategy for Nrf2 activation consists of reducing its β-TrCP mediated degradation, either directly with GSK-3 inhibitors or indirectly via activation of the PI3K/AKT pathway. The use of GSK-3 inhibitors prevents the kinase-mediated phosphorylation of Nrf2, which, in turn, precludes the binding of the transcription factor to the E3-ubiquitin ligase adaptor β-TrCP and its subsequent proteasomal degradation (Figure 5B and Figure 6) [207,208]. Consistent with the presence of elevated oxidative stress markers and decreased Nrf2 activity, GSK3-β was found to be hyperactive in the brain of AD patients [331,332]. This increased GSK3 signaling has been shown to be strongly associated with several AD neuropathological features, including tau phosphorylation, Aβ production, neurogenesis, memory impairment, and synaptic dysfunction [332]. Therefore, it is not surprising that GSK3 inhibition has emerged as a potentially important therapeutic approach for AD treatment. The inhibitors tested include, among others, the mood stabilizing drug lithium, FDA-approved for the treatment of epilepsy and bipolar disorders, the non-ATP competitive GSK-3β inhibitor tideglusib, the ATP-competitive specific GSK3 inhibitor SB216763, and the selective, brain permeable inhibitor AZD1080 [332,333,334,335,336,337]. All these inhibitors showed, in one way or another, proof-of-concept benefits in in vitro and in vivo models including increase in the anti-oxidant responses, decrease in tau phosphorylation and Aβ deposition, as well as amelioration of memory deficits in Tg mice [332,337,338,339]. Although in some cases encouraging benefits were observed in clinical settings, many problems were also encountered. These included toxicity issues, low efficacy, and severe off-target effects related, in part, to the ubiquitous expression of GSK3 in different tissues/organs, the multifactorial action of this protein kinase, and its role in the regulation of numerous vital cellular processes [332,335,340,341].

An alternative strategy for preventing Nrf2 binding to the β-TrCP complex for its proteasomal degradation is through targeting the PI3K/AKT pathway. Activation of this path leads to GSK3 phosphorylation, which renders the enzyme inactive for the Nrf2 phosphorylation that is required for the binding of the transcription factor to the E3-ligase complex (Figure 5B and Figure 6). This, in turn, prevents Nrf2 degradation and results in its accumulation, nuclear translocation, and subsequent activation of downstream target genes [191,341]. The PI3K/AKT activators investigated include many natural saponins and flavonoids such as gypenosides, sulfuretin, puerarin, and hesperidin which—although not as well studied as other compounds described above—provided benefits as antioxidants in various in vitro and in vivo settings [342,343,344,345].

A different group of compounds boosting Nrf2 signaling through the PI3K/Akt axis is constituted by agents with known antioxidant properties, encompassing various mechanisms and that only more recently have been described as Nrf2 inducers through this path. Among them are methazolamide (MTZ) and melatonin (MEL), two compounds with strikingly different primary activities, but which are capable of activating Nrf2 via the PI3K/GSK-3 path, through still not completely elucidated mechanisms [155,346,347,348,349,350].

MTZ is known to act as a carbonic anhydrase inhibitor that catalyzes the reversible hydration of carbon dioxide to produce bicarbonate and a hydrogen ion (CO_2_ + H_2_O ↔ HCO_3_^−^ + H^+^) and, as a result, it is capable of modulating physiological and pathological processes in which cellular pH buffering plays a relevant role [351,352]. Consistent with its ability to modulate CO_2_ concentrations, the compound has been reported to improve ventilation and oxygenation levels, properties that supported its use in the treatment of high-altitude sickness [353]. As it is the case with other carbonic anhydrase inhibitors, MTZ has also been employed in the treatment of epilepsy, a chronic brain disorder characterized by spontaneous recurrent seizures related to rapid changes in ionic composition, including increases in intracellular potassium concentrations and pH shifts [352,354,355]. The beneficial use of MTZ as an antiepileptic drug likely relates to the ability of the compound to generate hydrogen ions. This, in turn, may modulate potassium shifts and influence the function of proton-sensitive transmembrane proteins implicated in neuronal signaling including γ-aminobutyric acid type A receptors (GABAARs), N-methyl-D-aspartate receptors (NMDAR), H^+^-gated channels, and cation channels, overall reducing neuron excitability [352,356,357,358,359,360,361]. In a different context but also related to its activity as a carbonic anhydrase inhibitor, MTZ is capable of regulating the anaplerotic replenishing of TCA intermediates (Figure 2) and is essential for the regulation of carboxylating enzymes using CO_2_ as a substrate. Among these, malic enzyme, propionyl, methylcrotonyl-, and acetyl-CoA carboxylases are the most relevant in neurons [362].

MTZ has also been shown to exhibit carbonic anhydrase-independent functions through its anti-oxidative stress properties. Early studies searching a library of FDA-approved drugs identified MTZ as one of the compounds capable of protecting neuronal cells from H_2_O_2_ induced oxidative damage, increasing cell survival [363]. Years later, the ability of MTZ to inhibit ROS production in primary cortical neurons was correlated with the beneficial effects of the compound in inhibiting neuronal apoptosis, improving neurological behavior, and relieving cerebral edema following post-subarachnoid hemorrhage in mice [364]. In the AD field, research has demonstrated the ability of MTZ to counteract Aβ-mediated mitochondrial dysfunction. Accordingly, MTZ prevented the loss of mitochondrial membrane potential and the production of mitochondrial ROS, overall leading to the inhibition of caspase-mediated apoptotic pathways in different in vitro and in vivo models, while also protecting an APP Tg line from behavioral deficits [365,366,367]. A significant contributor to the effect of MTZ on the anti-oxidative stress response relates to its action on Nrf2. In this sense, the protective effect from high-altitude-induced cerebral vascular leak in rodent models was attributed, at least in part, to the capability of the compound to potently activate Nrf2 via PI3K activation [346]. Given the multifaceted properties of MTZ in targeting multiple interrelated pathways, it remains difficult to determine to what extent each of the different functions of the compound exert a more dominant role in its overall beneficial effect in neurodegenerative conditions.

Melatonin (MEL; N-acetyl-5-methoxytryptamine), is a neuro-hormone secreted by the pineal gland that regulates circadian rhythms of physiologic activities including sleep [368]. Recent findings have linked circadian clocks and sleep with neurodegeneration. This, together with the newly discovered role of sleep in the facilitation of the brain removal of Aβ and tau, along with that of other toxic proteins through the glymphatic system, have made the use of melatonin to promote healthy physical and mental aging and as a potential intervention in AD even more relevant [369,370]. In the context of this review, MEL was shown to act as a potent antioxidant that was active in different in vivo systems, including in the CNS and at the level of the synapses [371,372,373]. The compound is known to counterbalance oxidative stress and reduce cellular damage by directly scavenging H_2_O_2_, as well as hydroxyl (HO^•^) and superoxide anion (O_2_^•−^) radicals [374,375,376], decreasing free radical formation, and neutralizing reactive oxygen and nitrogen species. In turn, these properties ameliorate downstream effects on protein carbonylation as well as DNA and lipid oxidation in different experimental settings [377,378,379,380,381,382].

The beneficial role of MEL in reducing oxidative stress also takes place through its ability to activate Nrf2 via PI3K/GSk3 paths, inducing the nuclear translocation of the transcription factor and increasing expression of downstream antioxidant genes [383,384]. Consistent with these multiple properties, MEL treatment was shown to increase the levels of different endogenous antioxidants including catalase, superoxide dismutase, and glutathione peroxidase [385,386]. Recent work has also demonstrated a wider activity of MEL on mitochondrial function preserving mitochondrial membrane potential [387]—an important element for ATP generation and the maintenance of full mitochondrial function—and precluding astrocytic Aβ-mediated mitochondrial depolarization [372]. It was also reported to prevent caspase-3-mediated apoptosis and enhance ATP synthesis under conditions of metabolic- and radiation-mediated stress, overall improving mitochondrial energy metabolism [388,389,390]. Consistent with all these properties, MEL administration in different AD animal models ameliorated oxidative stress, memory deficits, and Alzheimer’s-related neuropathology including amyloid load, tau hyperphosphorylation, and neurodegeneration [377,384,390,391,392].

It should be mentioned that the overall beneficial effect of melatonin in AD extends beyond the antioxidant properties of the compound and its ability to regulate circadian rhythms. In this sense, it has been described that MEL is also capable of influencing the formation of Aβ by regulating the expression and activities of the secretases involved in the processing of the APP precursor protein [393]. In addition, MEL also exhibits a protective effect through its ability to interact with Aβ peptides and inhibit the progressive formation of β-sheets and amyloid fibrils, which in turn ameliorates the peptide neurotoxicity [378,393,394]. The multifaceted properties of MEL targeting different aspects of AD pathogenesis, while opening interesting avenues to explore, also make it difficult to dissect the underlying mechanisms responsible for the beneficial effects of the compound.

## 8. Conclusions and Future Directions

In spite of numerous advances in the investigation of different aspects of the pathobiology of AD, many unknowns remain regarding the complex molecular mechanisms triggering the disease and driving its progression. Multiple evidence indicates that detrimental changes in mitochondrial function with alterations in cell bioenergetics and disturbances of redox homeostasis that lead to oxidative stress conditions play a significant role in the disease pathophysiology, affecting numerous cellular pathways. The presence of oxidative stress markers, coinciding with increased levels of reactive oxygen and nitrogen species—a common finding with other neurodegenerative diseases—in early stages of the disease, and even preceding the neuropathological lesions, support a causative role in the disease. These findings, in turn, have fostered numerous studies using dietary supplementation with antioxidants, with the aim of delaying the onset and/or ameliorating the progression of the disease. However, despite varying success in different research settings encompassing cell culture and animal models, antioxidant trials have not provided significant protection in humans, reflecting, in part, limitations in the preclinical studies, short biological half-life, and/or limited bioavailability of the agents. More recently, research focus has shifted to the activation of endogenous antioxidant defenses through Nrf2, a central element modulating numerous cytoprotective genes and regulating not only the antioxidant response but also mitochondrial functioning and multiple points of the cell intermediary metabolism. The existence of diverse compounds capable of activating Nrf2 through different mechanistic pathways, disrupting its ubiquitin-dependent degradation by the 26S proteasome, and leading to Nrf2 nuclear accumulation and downstream gene induction, unveil novel promising therapeutic avenues. Exploiting the multifactorial effect of Nrf2 activation may not only contribute to restore cellular redox homeostasis, but also modulate the brain metabolic dysregulation present in AD. Indeed, it is currently becoming clear that successful prevention/delay of AD development will likely require complex strategies. Combination of antioxidant-rich diets with agents targeting multiple genes and pathways encompassing mitochondrial and synaptic function together with metabolic and bioenergetics regulation may help fine tune more definitive and precise therapeutic approaches.

## Data Availability

Not applicable.
